# Exploring the Frontier: The Human Microbiome’s Role in Rare Childhood Neurological Diseases and Epilepsy

**DOI:** 10.3390/brainsci14111051

**Published:** 2024-10-23

**Authors:** Newell Belnap, Keri Ramsey, Sophia T. Carvalho, Lexi Nearman, Hannah Haas, Matt Huentelman, Keehoon Lee

**Affiliations:** 1 Center for Rare Childhood Disorders, Translational Genomics Research Institute (TGen), Phoenix, AZ 85004, USA; nbelnap@tgen.org (N.B.);; 2 Translational Genomics Research Institute, Flagstaff, AZ 86001, USA; 3 Department of Biological Science, Northern Arizona University, Flagstaff, AZ 86011, USA; 4 TGen Integrated Microbiomics Center, Translational Genomics Research Institute (TGen), Flagstaff, AZ 86011, USA; 5 Barrett, the Honors College, Arizona State University, Tempe, AZ 85281, USA

**Keywords:** epilepsy, microbiome, gut–brain axis, drug-resistance epilepsy, rare childhood disease, microbiome-epilepsy, rare childhood neurological diseases

## Abstract

Emerging research into the human microbiome, an intricate ecosystem of microorganisms residing in and on our bodies, reveals that it plays a pivotal role in maintaining our health, highlighting the potential for microbiome-based interventions to prevent, diagnose, treat, and manage a myriad of diseases. The objective of this review is to highlight the importance of microbiome studies in enhancing our understanding of rare genetic epilepsy and related neurological disorders. Studies suggest that the gut microbiome, acting through the gut–brain axis, impacts the development and severity of epileptic conditions in children. Disruptions in microbial composition can affect neurotransmitter systems, inflammatory responses, and immune regulation, which are all critical factors in the pathogenesis of epilepsy. This growing body of evidence points to the potential of microbiome-targeted therapies, such as probiotics or dietary modifications, as innovative approaches to managing epilepsy. By harnessing the power of the microbiome, we stand to develop more effective and personalized treatment strategies for children affected by this disease and other rare neurological diseases.

## 1. The Human Microbiome: Basics and Beyond

“Microbiome” refers to the combined genetic material of all microbes within a specific niche, such as the human gut. The human body contains approximately 1.3 times more microbial cells (microbiota) than human cells and approximately 100 times more microbial genes than the human genome [1]. These microbial genes (the microbiome) encode a variety of functions that affect the digestive system, as well as overall health, through interactions with various parts of our body. The human microbiome, particularly the gut microbiome, which consists of billions of microorganisms, is now recognized to play a critical role in maintaining gut health, contributing to the maturation and function of the immune system and even influencing the function and homeostasis of other peripheral organs [2]. Research shows that the microbiome influences various conditions, from metabolic diseases like obesity and diabetes to mental health issues; our understanding of its impact throughout the body is constantly expanding [3,4,5]. 

### 1.1. Methodologies for Studying the Microbiome

The microbiomics field has made significant methodological advancements in recent years, and coinciding improvements in various technologies have underscored the field’s potential. The advent of next-generation sequencing (NGS) technologies and the development of advanced bioinformatics tools have revolutionized the study of microbiomes. These breakthroughs have, for instance, significantly reduced the time and cost required to analyze the entire gut microbiome, making it feasible to process the larger sample sizes necessary. As a result, large-scale cohort studies have become more accessible, leading to the discovery of associations between gut microbiota and various diseases, including brain disorders, obesity, diabetes, and inflammatory bowel disease. These advancements have greatly enhanced our understanding of the human gut microbiome and its impact on health [6]. Also, specific microbiome signatures associated with diseases and drug responses can be discovered through metagenomics, the study of genetic materials from the microbiome. The predominant sequencing methodologies for microbiome analysis include 16S rRNA amplicon sequencing, internal transcribed spacer (ITS) amplicon sequencing, and shotgun metagenomics. 16S rRNA amplicon sequencing is primarily used for bacterial profiling, while ITS sequencing is specific to fungal communities. These amplicon-based approaches are advantageous for samples with a low microbial load or high human DNA contamination, as they selectively amplify microbial-specific DNA regions. However, being PCR-dependent, they are susceptible to amplification-induced biases and artifacts, such as chimeric sequences and skewed microbial abundances. Additionally, these methods offer lower taxonomic resolution compared to shotgun metagenomics.

Shotgun metagenomics provides a higher taxonomic resolution, which is crucial for identifying disease-associated microbial biomarkers. It also enables the characterization of functional genes within the microbial community and is less prone to PCR-induced artifacts. Nonetheless, this method is more sensitive to issues in low-biomass samples and contamination from human DNA, as it does not rely on targeted amplification. To mitigate this challenge, strategies such as human DNA depletion prior to library preparation can be employed to improve the accuracy and efficiency of shotgun metagenomics in such contexts. 

Emerging technologies are poised to outdo even fully optimized shotgun metagenomics. Metatranscriptomics allows for the study of gene expression in gut microbes related to diseases, thereby identifying the specific metabolic pathways involved. Additionally, multi-omics approaches enable the simultaneous analysis of a patient’s gut microbiota and metabolome, a set of small molecules that exist in a sample. This can reveal the association of specific microbial communities with drug responses, providing critical information for developing personalized treatment strategies [7,8].

In the future, the continuous evolution of these technologies will further deepen microbiome research and significantly enhance our understanding of human health and diseases.

### 1.2. Composition and Function of the Human Microbiome

The human microbiome begins to develop immediately after birth, gradually stabilizing by the age of three [7]. This established microbiome contributes to maintaining the body’s homeostasis. It is estimated that approximately 50 different bacterial phyla exist in the gut microbiota, with six being major players: Actinobacteria, Proteobacteria, Fusobacteria, Verrucomicrobiota, Bacteroidetes, and Firmicutes; Bacteroidetes and Firmicutes comprise > 90% of the total flora population in the gut [9]. The disruption of the microbiome’s composition, termed dysbiosis, is increasingly recognized as a factor in various diseases. For example, compelling evidence links gut microbiota to cardiovascular health. Short-chain fatty acids (SCFAs) produced by beneficial bacteria like *Bifidobacterium* and *Faecalibacterium prausnitzii* regulate blood pressure and cholesterol, and a decrease in these bacteria is associated with a higher risk of cardiovascular disease (CVD) [10,11]. The gut microbiome also influences energy metabolism. A high abundance of Firmicutes bacteria, known for their efficient energy extraction, is linked to obesity and type 2 diabetes. Conversely, a higher abundance of Bacteroidetes, which promote leanness, is associated with a healthier metabolic profile [12]. Furthermore, dysbiosis in the gut microbiome plays a role in inflammatory bowel disease (IBD). A decrease in beneficial bacteria like *Bifidobacterium* and *Lactobacillus*, coupled with an increase in potentially harmful bacteria like *E. coli*, can disrupt the gut epithelial barrier and trigger chronic inflammation [13]. These relationships emphasize the critical role of the microbiome in human health. Understanding them opens new avenues for developing novel and tailored therapeutic strategies, such as fecal microbiota transplants (FMTs), to prevent and manage numerous diseases.

### 1.3. The Microbiome’s Influence on the Immune System

The microbiome also plays an important role in immune system development and function at all stages of life. During pregnancy, metabolites and microbiome-induced antigens produced by the mother’s microbiome can cross the placenta and reach the fetus. This exposure helps the fetus adapt its immune system to the mother’s microbial environment, preparing it for birth, when the mother’s microbiome will colonize the infant’s gut [14]. This exposure is not only tolerated but also required for proper immune development [15]. Throughout the first three years of life, the child’s microbiome undergoes dramatic changes influenced by diverse factors, including diet, geographic location, medical care, etc. As the innate immune system matures, the microbiome stabilizes [15,16,17]. The microbiome continues to shape the immune system throughout life. It acts as a training ground for immune cells, educating them to differentiate between harmless microbes and potential pathogens. This delicate balance is crucial for preventing chronic inflammatory diseases and allergies [18,19]. Additionally, the microbiome produces beneficial metabolites that influence immune function and regulate inflammation. In essence, the microbiome acts as a key partner in the immune system’s development and ongoing regulation [20].

## 2. Rare Childhood Neurological Diseases

There are more than 10,000 unique rare diseases, which have environmental, infectious, cancer, and gene-based etiologies. Nearly half of all rare diseases are neurological, affecting the brain, spinal cord, and/or peripheral nerves and muscles. Among these, genetic etiologies predominate: 80–85% of rare neurological conditions can be traced to pathogenic variants in single genes that disrupt the functions of essential proteins [21]. Rare diseases are defined by prevalence, usually averaging about 40–50 cases per 100,000 people. Despite the low prevalence, the cumulative impact of rare diseases is substantial, with more than 33 million people affected in the United States and more than 300 million people affected worldwide [22]. 

Advancements in genomic technologies have improved diagnostic timelines and rates; yet, individuals living with a rare disease typically spend more than five years searching for a diagnosis, and up to 50% remain undiagnosed [23,24,25]. Furthermore, less than 5% of rare diseases have FDA-approved treatment options [23]. The current standard-of-care for the majority of rare diseases is symptomatic management only. As a consequence, many patients live with life-long disabilities. Rare diseases disproportionately affect children, and early mortality is tragically high, with 3 in 10 not living to see their fifth birthdays [22,25]. 

An accurate diagnosis is recognized as “central to the practice of medicine” and “essential for informed care and promoting patient and family well-being” [23,24]. Nevertheless, individuals and families with a rare neurological disease frequently encounter multiple challenges in receiving an accurate diagnosis. This process is so commonplace that it is routinely referred to as a diagnostic odyssey and is usually filled with physical, mental, financial, and emotional stressors. Diagnosing and treating rare diseases poses unique challenges to both families and physicians that are not typically present when dealing with more common illnesses.

Considering the serious unmet medical needs and difficult circumstances faced by patients and their families, further research into various rare diseases is strongly encouraged [26]. Because of the profound influence the gut microbiota have on the nervous system and overall health, it is imperative that we as a field explore this complex mechanism and its relation to rare childhood neurological disorders as a means to improve diagnostic rates and provide potential treatment options [27].

## 3. The Microbiome and Neurological Health: Establishing the Connection

Over the past two decades, the fields of microbiology and neurology have come together to study an incredible aspect of human health: the gut–brain axis. This bi-directional communication pathway connecting the immune, endocrine, and central nervous (CNS) systems is essential for the maintenance of normal body function and health. Dysbiosis of the microbiome can therefore harm other regions of the body, and numerous studies have demonstrated the influence of microbiota on the nervous system [27,28]. For instance, distinct differences are seen in the gut microbiota composition of children with autism spectrum disorder (ASD) compared to that of neurotypically developing children. These differences are believed to influence neurodevelopment and behavior through the gut–brain axis [29]. In an example of beneficial microbial influence, a fecal transplant study of children with ASD and their healthy siblings demonstrated significant improvement in ASD symptoms in the transplant recipients, suggesting that modifying the gut microbiota could benefit individuals with ASD—again, through the gut–brain axis [30,31]. 

A systematic review of gut microbes’ impact on neurodevelopment by Caputi et al. examined alterations in the intestinal microbiota of individuals with neurodevelopmental disorders such as ASD, ADHD, and Rett syndrome. The reviewers highlighted the functional contributions of the gut microbiome, pointing to significant alterations that could cause or exacerbate the symptoms and challenges associated with these disorders [32]. This phenomenon could be particularly critical in the earliest stages of life, as alterations in the microbiome during those periods have been linked to an increased risk of developing neurodevelopmental disorders later in life. For example, antibiotic use in infancy has been associated with disrupted gut flora and a heightened risk of developing neuropsychiatric disorders later in life [5,33].

### 3.1. Epilepsy

Epilepsy is a debilitating, chronic, heterogeneous disease of the brain that afflicts nearly 70 million people worldwide, with a considerable social and economic burden. Epilepsy is the most frequent chronic neurologic condition in childhood and affects 0.5% to 1% of children; it is characterized by transient clinical manifestations arising from synchronized, high-frequency, or excessive abnormal neuronal activity in the CNS, which results in temporary neurological dysfunction [9,34]. The prevalence of epilepsy does not differ by gender or age group [35,36]. 

Epilepsy leads to a diminished seizure threshold and the predisposition to have repeated seizures, which affect the “neurobiological, cognitive, psychological, and social consequences of this condition” [35,37]. Epilepsy is diagnosed when an individual has two unprovoked seizures occurring more than 24 h apart, has a single unprovoked seizure (if the recurrence risk is high), or has a prior diagnosis of any epilepsy syndrome [38].

Seizures are required for the diagnosis of epilepsy, but not all people who experience a seizure have epilepsy. Seizures are episodes of abnormal, excessive, hypersynchronous neuronal activity in the brain that lead to uncontrolled alterations in neurologic function. They present as objective signs or subjective symptoms such as decreased awareness, involuntary muscle contractions, an unusual smell, involuntary laughing, and stiffening. Seizures develop when the excitability of cortical neurons exceeds a certain threshold [37,39,40]. In 2017, the International League Against Epilepsy (ILAE) revised the classification of seizures into focal (seizures arising in one hemisphere of the brain), generalized (seizures originating in both hemispheres simultaneously), and unknown onset, with subcategories that include motor and non-motor. Focal seizures can be further categorized by consciousness status with retained or impaired awareness. The most common types of epilepsy are generalized and unknown, characterized by seizures of the same categories [41].

The diverse pathophysiology of epilepsy corresponds to its equally diverse etiologies. Identifying the underlying etiology of epilepsy is vital for effective treatment and optimal prognosis [35,37]. In the ILAE classification, etiologies are divided into six categories: structural, genetic, infectious, metabolic, immune, and unknown. These groupings are not mutually exclusive, and many etiologies fall into more than one category. The relative prevalences of each category observed within a geographic region vary; for example, least developed countries (LDC) often have higher rates of infection-related epilepsy [35,37,42].

### 3.2. The Microbiome–Epilepsy Link

The pathogenesis of epilepsy is multifactorial, and the specific mechanisms are unknown. Until recently, it was believed that the etiology of most neurological disorders was related to abnormal brain function. Numerous animal and clinical studies now suggest that the microbiota via the gut–brain axis have a role in multiple neurological disorders, including epilepsy [9,43]. Studies in animal models have shown multiple potential mechanisms that can influence epileptogenesis. The gut microbiome has a critical role in maintaining the integrity of the blood–brain barrier (BBB), which, when compromised, leads to neuroinflammation. SCFA produced by the GM have been shown to upregulate the expression of tight junction proteins, which are important for BBB permeability. The GM also helps regulate the production of glutamine and GABA, which is important in maintaining the proper balance in excitation/inhibition for neuronal synapses. *Akkermansia muciniphila* and *Lactobacillus acidophilus* can augment the endocannabinoid system in controlling neuronal excitability [44].

Other studies of the gut microbiome have demonstrated that certain gut bacteria can produce compounds that affect the central nervous system, potentially influencing seizure susceptibility and frequency [45]. Ketogenic diets, which alter the gut microbiome, have been used effectively to manage drug-resistant epilepsy (DRE), the “failure of adequate trials of two tolerated and appropriately chosen and used anti-epileptic drug schedules (whether as monotherapies or in combination) to achieve sustained seizure freedom”, in children, indicating a possible link between diet-modulated microbiome changes and seizure control [9,43,46,47]. These studies contribute to a growing body of evidence suggesting that the gut microbiome plays a crucial role in the development and manifestation of neurodevelopmental disorders, including epilepsy. The microbiome’s influence on the central nervous system, possibly through the gut–brain axis, points to new directions for research, prevention, and treatment strategies for these conditions.

Additionally, multiple population-based studies revealed differences in gut microbiota between healthy controls and patients with epilepsy [9]. Xie et al. reported that 14 infants with refractory epilepsy had a gut microbiome composition that differed significantly from that of healthy controls (*n* = 30), with lower alpha diversity and a significant increase in Firmicutes and Proteobacteria [46]. Similarly, Peng et al. reported that refractory epilepsy (*n* = 42) patients exhibited a markedly different microbiota composition from that seen in people without the disease, including an increased prevalence of typically rare phyla like Verrucomicrobia and increased alpha diversity when compared with patients with non-refractory epilepsy (*n* = 49). Increased seizure frequency and lowered alpha diversity also correlated with decreased populations of *Bifidobacterium* and *Lactobacillus* [48].

Furthermore, Şafak et al. compared 30 patients with focal epilepsy to 10 non-epileptic healthy controls using principal component analysis and demonstrated that the composition of gut bacteria clustered differently in patients with epilepsy. Higher Proteobacteria and lower Fusobacteria were observed in the epilepsy group [49]. 

Studies by Dong et al. reinforced these results, again uncovering significantly increased Fusobacteria. Dong et al. also concurred with Xie et al., reporting significant differences in the gut microbiota composition between epilepsy patients and non-epileptic healthy controls. *Fusobacterium mortiferum*, *Bacteroides fragilis*, *Ruminococcus gnavus*, and *Fusobacterium* spp. were identified as potential risk factors for developing epilepsy, with increased levels of *Fusobacterium* in particular standing out as a potential diagnostic biomarker for epilepsy [50,51].

Ouyang and colleagues explored the potential causal relationship between the gut microbiota and epilepsy and identified specific microbe taxa that were associated with three subtypes of generalized epilepsy. They collected genetic variants from a previous genome-wide association study, gut microbiota, and metabolites from the gut microbiota to conduct a bi-directional Mendelian randomization study. They found that the family Veillonellaceae (phylum Firmicutes) was associated with a higher risk of childhood absence epilepsy. Class Melainabacteria was coupled with a lower risk of generalized epilepsy with tonic-clonic seizures; and class Betaproteobacteria, along with the order Burkholderials (phylum Proteobacteria), are potential protective factors in juvenile myoclonic epilepsy [9,52].

Studies of DRE by Peng et al. and Lee et al. showed similar results, with an increase in phylum Firmicutes. Previous studies of the phylum Proteobacteria in relation to epilepsy are less consistent [48,52,53]. These inconsistencies may result from differences in the study design, sample size, or analysis method. Despite these discrepancies, a growing body of preclinical and clinical studies support the idea that patients with epilepsy often display alterations in their gut microbiome. Moreover, patients with DRE may experience even more changes in the composition of their microbiota when compared to non-epileptic controls and patients with drug-sensitive epilepsy [54].

### 3.3. Potential Microbiome Mechanisms in Epilepsy Pathogenesis

The underlying mechanisms of how the gut microbiota are related to epilepsy are still not fully understood. The gut microbiota interact with the brain primarily through the nervous, endocrine, and immune systems and through metabolic signaling pathways. Furthermore, the influence of the gut microbiome on epilepsy may not rely on one specific pathway and instead on a combination of all these pathways [52]. As studies have shown, patients diagnosed with epilepsy experience an increase in the bacteria phyla Firmicutes, Proteobacteria, and Verrucomicrobiota, all of which are immune-harmful, whereas Bacteroidetes and Actinobacteria, which are immune-beneficial, are decreased (Table S1) [36,45].

**Figure 1:**
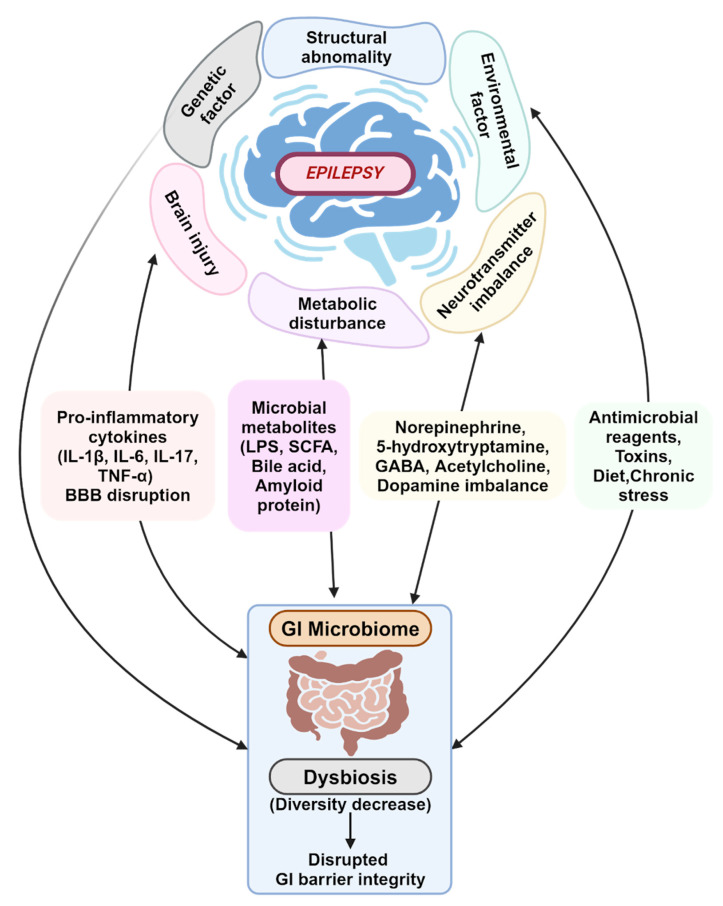
The Intricate Web between the Gut Microbiome and Epilepsy. The gut microbiome and its contributions to epilepsy progression are influenced by numerous internal and external factors. Created in BioRender. Lee, K. (2024). www.BioRender.com/v29e118, accessed on 6 October 2024.

Epileptogenesis has been linked to neuroimmunity and neuroinflammation. The gut microbiota impact the inflammatory and immune pathways by regulating the maturation of microglial cells and activating astrocytes (Figure 1). These nerve cells are vital to the immune and inflammatory responses of the CNS [36]. Astrocytes and microglia interact in a way that leads to increased pro-inflammatory cytokine production and BBB permeability. This causes the intrusion of peripheral blood immune cells and cytokines into the CNS and subsequent chronic neuroinflammation (Table S1) [36,45].

The gut microbiota are pivotal in maintaining the integrity of the BBB. The gut microbiota can induce glial cells, including microglia, to release pro-inflammatory cytokines that induce neuronal hyperexcitability and, eventually, seizures. The permeability of the BBB is also susceptible to inflammatory cytokines secreted by gut bacteria into systemic circulation; these activate peripheral immune cells, which can promote neuroinflammation and increase neuronal excitability, also ultimately resulting in seizures (Figure 1, Table S1) [45,50]. In a study by Liu et al., it was demonstrated that mice lacking gut microbiota display increased BBB permeability, which was associated with disorganized tight junctions. Remarkably, this could be rescued by recolonization with gut microbiota or supplementation with short-chain fatty acids (SCFAs), emphasizing the crucial role of the gut microbiome in maintaining the integrity of the BBB and preventing neuroinflammation [55].

Short chain fatty acids (SCFA) produced by the gut microbiota can influence the immune system by stimulating the synthesis and release of immunoglobulins from plasma cells. It is theorized that SCFAs are also able to exert neuromodulatory effects, such as decreasing seizure intensity and increasing the threshold for seizures (Figure 1). When dysbiosis occurs, a decreased production of SCFA results, negating these neuroprotective effects (Table S1) [45,50]. Another study by Li et al. demonstrated that the supplementation of sodium butyrate decreased the seizure intensity and had a neuroprotective effect in terms of hippocampal neuron loss following seizures, further highlighting the involvement of SCFAs in seizure activity and brain health [56].

Additionally, gut microbes may be able to regulate the metabolism of dietary tryptophan, which can lead to the release of pro-inflammatory cytokines and neurotoxic metabolites and the development of chronic neuroinflammation and seizures (Figure 1). Neurotransmitter imbalance has been indicated as one of the major causes of epilepsy. Certain gut microbes secrete neurotransmitters like norepinephrine, 5-hydroxytryptamine, GABA, acetylcholine, and dopamine. These are important to the regulation of the immune response and crucial for homeostasis and can directly or indirectly affect the excitability of neurons in the CNS, promoting seizures (Figure 1, Table S1) [36,45,50]. 

It is known that stress can promote the induction of epilepsy. The hypothalamic–pituitary–adrenal (HPA) axis is a neuroendocrine system central to the stress response [57]. Different hormones from the HPA evoke different seizure effects. Most deoxycorticosterones exert anti-seizure effects, while the corticotropin-releasing hormone and corticosterone may promote seizures. Evidence has suggested that gut microbiota can influence the HPA, although the specific mechanism is not known. It has further been proposed that stress can upregulate glucocorticoids and heighten glutamatergic signaling to promote seizures. Cortisol receptors are expressed on many types of cells in the gut and can also affect the functionality of the intestines, thus altering the microbiota (Table S1) [36,58]. 

The microbiota can impact the CNS directly, through the vagus nerve, or indirectly, by way of the enteric nervous system (ENS). Enteroendocrine cells (EECs) found in the lumen of the intestines are gut sensory epithelial cells capable of eliciting neurotransmission in response to external stimuli. These cells have an assortment of molecular receptors that can detect microbiota components, catabolites, and toxins. EECs can sense stimuli secreted by the GM which interact with various neurotransmitters and conduct signals through the vagus nerve, which can affect CNS excitability (Table S1) [9,59]. 

Neuropod cells are a specialized subtype of EECs that synapse with the vagus nerve and transduce sensory signals from the lumen of the intestines to the brainstem. Neuropod cells play a critical role in sensing metabolites produced by the gut microbiota, including SCFAs, bile acids, phenols, indoles, bioactive lipids, and neurotransmitters (Table S1) [9,60]. 

### 3.4. Microbiome-Related Epilepsy Treatment

Anti-epileptic drugs are the current standard of care for epilepsy management. This treatment modality is successful in treating this chronic condition for a majority of patients, but up to 30% of patients suffer from DRE [43,45,61]. Surgery, vagus nerve stimulation, and the ketogenic diet (KD) are options for individuals with DRE. The KD has been a treatment option for epilepsy since the 1920s, with some studies reporting that more than half of patients adhering to the diet see a 50% reduction in seizures, one-third see 90% seizure reduction, and some patients achieve complete control of their seizures [40,43]. Clinical studies and animal models reveal the potential role the gut microbiota have in DRE. Patients with refractory epilepsy have a higher alpha diversity compared to drug-sensitive patients [9]. Olson and colleagues performed studies in germ-free mice to better understand the mechanism by which the KD decreases seizures. Although some bacterial species were lost in response to the KD, the authors did find a decrease in alpha diversity and an increase in *Akkermansia muciniphila*, *Parabacterioides*, *Sutterella*, and Erysipelotrichaceae. These studies altered the abundance of specific bacterial species and demonstrated that adherence to the KD altered the microbiome in such a way as to increase the GABA/glutamate ratio levels and thereby decrease seizures. Their research demonstrated that seizure improvement arising from the KD was induced by the augmentation of selective gut bacteria. The underlying mechanism hinges on microbial interactions that decrease bacterial glutamyltranspeptidase activity, which results in decreased gamma-glutamylated amino acids, which in turn affects the GABA/glutamate ratios in the hippocampus [43].

## 4. Future Research Approaches

### 4.1. Emerging Technologies for Microbiome Research

Existing technologies used in microbiome research are being continually refined for improved accuracy and higher throughput. The difficulties associated with microbe identification via culturing in early research development spurred sequencing technology advancements and the identification of many bacterial species through genomic methods [62]. The transition from traditional culturing techniques to first-generation DNA sequencing (utilizing the Sanger method) paved the way for the current use of second-generation sequencing. Divisions between the emerging third and fourth sequencing generations remain the subject of much discussion; so far, the implementation of single-molecule sequencing and nanopore technology, generally considered third-generation sequencing techniques, is becoming more widespread [63,64]. Both techniques offer long-read sequencing without concern for amplification biases [65]. The long-read sequencing technique allows for the more accurate assembly of microbial genomes and a better understanding of the overall structure of the genome, including complex repetitive sequences and structural variations. Long-read sequencing significantly improves the accuracy and resolution of taxonomic identification within the microbiome, which helps us identify the functional genes of microbes and better elucidate their metabolic pathways [66]. This plays a crucial role in improving the prevention, diagnosis, and treatment strategies for various diseases. Nanopore technology, in particular, enables single-molecule sequencing (when charged biological molecules pass through nano-scale holes) [64]. This combination of single-molecule and long-read sequencing strategies offers significant prospects as a genetic diagnostic tool. Beyond real-time targeted sequencing, nanopore technology can detect structural variants in genomes, which are commonly observed in cancer [67]. Single-molecule sequencing, with an emphasis on nanopore technology, presents a prominent emerging toolkit that can greatly contribute to the diagnosis of genetic conditions, particularly those impacted by the microbiome and dysbiosis. 

Cutting-edge advancements are not limited to sequencing technologies. Microbiome multi-omics strategies include culturomics, metatranscriptomics, and metabolomics, which are all primarily used to investigate samples with a lower microbial biomass [62]. As low-biomass organs are difficult to characterize in microbiome ecology, the use of high-throughput cell cultures, metagenomic mRNA analysis, and metabolite identification via multi-omics is invaluable in the identification and analysis of the small quantities of microbes present in these organs. After microbes are identified and their functionality hypothesized, investigations of microbiome changes are often completed in vitro or within animal models. While these approaches remain largely reliable and serve an important role within microbiome analysis and understanding, the emerging use of organ-on-a-chip and host-microbiota module technology allows researchers to better assess human host responses independently of traditional models. One such gut-on-a-chip model is HuMiX, which is a modular, microfluidics-based, human-microbial co-culture model that incorporates a separatory nanoporous membrane between the human cells and bacteria [68]. Effective models of the GI human–microbiome interface are essential to researchers’ efforts to elucidate the microbial mechanisms of disease, and groundbreaking technologies like HuMiX highlight the continuing progress of the microbiome field toward such goals. These advances in microbiome research technologies and methodologies have revealed the increasing relevance of the gut–brain axis and its potential role in personalized medicine.

### 4.2. The Potential of Microbiome Profiling and Personalized Medicine in Treating Rare Neurological Diseases

Commonalities in gut microbial compositions across individuals can help us to identify common disease indicators, especially when broad patterns of dysbiosis are present. However, detecting rare diseases often requires a more detailed and precise analysis of the gut microbiome, as the microbial changes involved may be subtle or unique [69]. There is a dire need for alternative diagnostic strategies, such as the detailed analysis of the gut–brain axis and microbial mechanisms, to potentially uncover biomarkers that genomic sequencing missed [23]. An understanding of the specific roles that differing strains of each microbe play in the pathogenesis and progression of disease is necessary to enable the identification of diseases which have heretofore evaded diagnosis. It may also be important to understand if and how an individual’s underlying germline genetic changes affect their microbiome. For instance, seizures and severe constipation, which often lead to a shift in the balance of the gut microbiome and metabolism, are major findings in Rett syndrome and CDKL5 deficiency disorder, caused by variants in the *MECP2* and *CDKL5* genes, respectively [70]. These genetic mutations may influence both neurological and gut health, suggesting a potential bidirectional relationship between the host’s genetic makeup and the composition of their microbiome. Identifying unique disease-associated microbiome profiles may assist in diagnosing currently undiagnosed individuals or solidify a diagnosis in individuals with variants of uncertain significance. Even seemingly minute differences in the gut microbiome may be underlying factors in rare diseases and must be considered and amended. After diagnosis, therapies such as prebiotics, probiotics, postbiotics, symbiotics, and fecal microbiota transplantation can be implemented to directly address microbial dysbiosis. Personalized therapies have the potential to impact the treatment of rare diseases by targeting the differences in microbial presence and/or abundance that are particular to that individual. Due to the prominence of the gut–brain axis, the treatment of rare neurological diseases is an area of particular interest that can be targeted via the gut microbiome. 

In a study by Su et al., multikingdom (archaea, fungi, and viruses) and gut microbiota markers were used to help diagnose ASD [71]. This study involved the metagenomic sequencing of fecal samples from 709 children with ASD and 374 neurotypical children. Children with ASD were found to host decreased levels of *Streptococcus thermophilus* and SCFA-producing bacteria (*Bacteroides* spp. PHL2737 and *Lawsonibacter asaccharolyticus*). Using a matched cohort of an additional 602 individuals with and without ASD, the team demonstrated that their novel model of 31 microbial features can facilitate the diagnosis of ASD [71]. Similar studies in epilepsy could provide valuable new tools for predicting therapeutic responses to anti-epileptic medications and even assist clinicians in anticipating the possible development of refractory epilepsy in their patients.

### 4.3. Prospects for Microbiome-Based Interventions and Therapeutic Strategies in Epilepsy and Other Neurological Conditions

Approximately 30% of epilepsy patients have refractory seizures which cannot be controlled by anti-epileptic medications [72]. While the precise mechanism of refractory epilepsy remains unknown, improvements in seizure symptoms are a promising step toward treating this neurological condition via the gut microbiome. Additional research into related treatments, such as fecal microbiota transplantation, attempts to more directly address the gut dysbiosis observed in epilepsy. As an example, in a study of patients with ASD, Kang et al. reported a 45% decrease in ASD symptoms (affecting language, social interaction, and behavior) two years after undergoing Microbiota Transfer Therapy (MTT), a type of fecal transplant, from a donor without ASD [73]. As microbiome research technologies evolve and our understanding of the gut–brain axis continues to improve, microbiome-based interventions can be anticipated to grow in prevalence and efficacy in the clinical setting.

## 5. Challenges and Considerations

### 5.1. Ethical, Technical, and Methodological Challenges in Microbiome Research

Microbiome research comes with unique ethical considerations. Unlike germline genetic studies in rare childhood neurological disorders, for which established regulations and professional etiquette exist, microbiome studies do not yet address the same concerns for patient safety and privacy. The gut microbiome is more dynamic than the nervous system, changes as we age, and is susceptible to a tremendous degree of natural variation, including intraindividual variation (variation between samples taken at different times from the same person). It is this ability to change that drives research into therapeutic interventions. This mutability could also fuel patient safety concerns in the future. Within the microbiomics field, the identification of secondary findings and struggles with genetic insurance discrimination are minimal, if non-existent, at this time. However, if this technology evolves into a major means of diagnosis and treatment, the regulatory landscape will also need to evolve, requiring commensurate testing and reporting standards. Chuong et al. suggested that microbiome results should be returned to patients if they are “analytically valid, reveal an established and substantial risk of a serious health condition, and are clinically actionable” [74]. Without regulations or established guidelines, determining risk or clinical relevance complicates reporting [75].

Determining the profile of a “healthy” reference microbiome, or identifying optimal control populations for microbiome studies, is another challenge. Larger studies have shown a substantial difference in the microbiome of healthy individuals, and the causes of such variance are currently unknown [76]. What is known is that an individual’s profile is influenced by their genetics, environmental and lifestyle factors, ethnicity, geographic location, and diet. For instance, in non-industrialized countries, gut microbiota are typically geared toward fiber degradation; in industrialized countries, they reflect a profile tailored to mucin degradation and exposure to medications and antibiotics [76].

Through the gut–brain axis, the gut microbiome’s influence on the nervous system may extend to mental and behavioral health. In a study by Zheng et al., germ-free mice were colonized with pooled fecal samples from individuals with non-medicated major depressive disorder [77]. Behavioral testing two weeks post-transplantation showed increased depression-like and anxiety-like behaviors when compared to animals with a “healthy microbiota” transplant. Additional studies in animal models have also seen similar findings [78,79]. While short-term adverse events, such as gastrointestinal issues of bloating, gaseousness, and changes in bowel habits, as well as long-term events, such as obesity and immune-mediated disorders, have been reported in FMT individuals, the potential benefits of such treatments mean that the long-term analysis of behavioral and mental health effects in humans is warranted [80]. 

### 5.2. Considerations for Patient Safety, Privacy, and the Regulatory Landscape

Due to the ever-growing popularity of personalized medicine and direct-to-consumer testing, over thirty companies worldwide now sell products designed to assess “gut health” [81]. Many of these companies advertise the ability to identify imbalances in gut health and provide customized recommendations for diet, probiotics, prebiotics, and supplements. Several companies further tout the ability to promote weight loss and assist in optimizing blood sugar levels. Some argue that these tests require more regulation, as they lack clinical validation and can financially exploit the general public; the results generated by many commercial tests are difficult for even doctors to interpret [81,82]. Furthermore, methods of collection, processing, analysis, and reporting are not standardized. In a study by Forry et al., they compared 16S and whole genome sequencing results from 44 academic, commercial, and government labs using seven shared reference samples. They found that protocol choices have significant effects on results, and significant and systematic measurement bias was also present [83]. 

While there are no FDA-approved clinical microbiome tests, over 1200 actively recruiting gut microbiome clinical trials are listed on clinicaltrials.gov (as of August 2024). Eight of these studies focus on epilepsy, including the effects of the ketogenic diet, probiotics, vagal nerve stimulation, and fecal microbiota transplantation; one focuses specifically on the genetic CDKL5 disorder [84]. An international and multicenter study named GEMMA (Genome, Environment, Microbiome, and Metabolome in Autism) is currently investigating the microbiome of around 600 siblings of individuals diagnosed with ASD from birth through the first three years of life [85]. The study hopes to identify biomarkers that will provide a better understanding of the development of ASD in children and enable the early diagnosis and treatment of ASD and related gastrointestinal symptoms. Similar large-scale studies in epilepsy may be needed to have a significant impact in the field.

## 6. Conclusions

The human microbiome is intricately and intrinsically linked to all body systems, playing a critical role in maintaining overall health. Its influence extends beyond the gut, reaching through complex interactions into the immune, endocrine, and nervous systems. As such, dysbiosis serves as a significant clue in understanding a myriad of diseases and disorders, including neurological conditions. This review has summarized the growing body of evidence that connects the microbiome to neurological diseases, ranging from common conditions like ASD to rare childhood disorders such as epilepsy.

The value of studying the microbiome in neurology, particularly in epilepsy, is becoming increasingly clear. New research suggests that specific microbial community profiles influence neurological development and seizure activity, providing potential avenues for novel microbiome-targeting therapies. As technological advances continue to improve our ability to analyze and understand these intricate microbial ecosystems, answers to longstanding questions about disease mechanisms and treatment options are becoming more and more accessible.

While the microbiome field as a whole is advancing rapidly, we are still only beginning to understand the full scope of the microbiome’s specific role in neurological health. The potential for future research is vast but not without its challenges. Methodological limitations such as sample variability and the complexity of microbial community–host interactions are obstacles that urgently need to be overcome.

As research continues to delve into the microbiome’s influence on the brain, it is clear that this field holds great potential for unlocking novel insights into the pathogenesis and treatment of neurological disorders, particularly those affecting children. Ultimately, by integrating microbiome study with neurology, we may be able to offer new hope for early diagnosis, improved outcomes, and better quality of life for the patients and families impacted by these challenging conditions.

## Data Availability

No new data were created.

## Supplementary Materials

The following supporting information can be downloaded at: https://www.mdpi.com/article/10.3390/brainsci14111051/s1, Table S1: Potential microbiome mechanisms in epilepsy pathogenesis.
