# Supplementary material for: Exploring the Frontier: The Human Microbiome’s Role in Rare Childhood Neurological Diseases and Epilepsy
Source: Brain Sci. 2024 Oct 23;14(11):1051. doi: 10.3390/brainsci14111051 (PMC11592123; doi:10.3390/brainsci14111051)
Supplement: Supplementary file 1 [file brainsci-14-01051-s001.zip › brainsci-3271427-supplementary.pdf]

Table S1. Potential microbiome mechanisms in epilepsy pathogenesis

**Mechanism**	**Description**	**References**
Gut microbiota composition changes	Increase in Firmicutes, Proteobacteria, Verrucomicrobiota (immune-harmful), and decrease in Bacteroidetes, Actinobacteria (immune-beneficial).	[36, 45]
Neuroimmunity and neuroinflammation	Gut microbiota impacts maturation of microglia and astrocyte activation, promoting neuroinflammation and seizure activity.	[36]
Blood-brain barrier (BBB) permeability	Increased BBB permeability due to pro-inflammatory cytokines, allowing peripheral immune cells into CNS and increasing neuroinflammation.	[36, 45]
Short chain fatty acids (SCFAs) imbalance	SCFAs, produced by gut microbiota, reduce seizure intensity and increase seizure threshold. Dysbiosis reduces SCFA production, negating these effects.	[45, 50]
Tryptophan metabolism	Gut microbes regulate tryptophan metabolism, leading to release of pro-inflammatory cytokines and neurotoxic metabolites, resulting in seizures.	[45, 50]
Neurotransmitter imbalance	Certain gut microbes secrete neurotransmitters (e.g., GABA, dopamine) that affect neuron excitability and promote seizures.	[36, 45, 50]
Stress and HPA axis	Stress influences the HPA axis, altering microbiota and potentially promoting seizures through cortisol and glutamatergic signaling.	[36, 56]
Direct impact via Vagus nerve	Microbiota can affect the CNS directly via the Vagus nerve, influencing brain excitability and seizure activity.	[9, 57]
Enteroendocrine cells (EECs)	EECs detect microbiota components and signal through the Vagus nerve, affecting CNS excitability.	[9, 57]
Neuropod cells	Neuropod cells sense microbiota metabolites and transduce signals to the brainstem, impacting CNS excitability.	[9, 58]
